# Compliance among infants exposed to hepatitis B virus in a post-vaccination serological testing program in four provinces in China

**DOI:** 10.1186/s40249-019-0568-y

**Published:** 2019-07-04

**Authors:** Hui Zheng, Guo-Min Zhang, Po-Lin Chan, Fu-Zhen Wang, Lance Everett Rodewald, Ning Miao, Xiao-Jin Sun, Zun-Dong Yin, Jeffrey Edwards, Hua-Qing Wang

**Affiliations:** 1 National Immunization Programme, Chinese Center for Disease Control and Prevention, Beijing, China; 2 HIV, Hepatitis and STI, WHO Regional Office for the Western Pacific, Manila, Philippines; 3 Department of Global Health, University of Washington, Seattle, Washington USA

**Keywords:** Post-vaccination serological testing, Operational research, Prevention of mother-to-child transmission, Vertical transmission, Loss to follow up, Disease elimination

## Abstract

**Background:**

Mother to child transmission of hepatitis B virus (HBV) remains the most common form of HBV infection in China. Prevention of HBV vertical transmission involves timely administration of the complete hepatitis B vaccine (HepB) series and hepatitis B immunoglobulin. Post-vaccination serological testing (PVST) is utilized to determine an infant’s outcome after HBV exposure and completion of HepB series. We aim to determine the frequency of compliance with a PVST testing cascade for HBV infected mothers and analyze factors associated with infant lost to follow up (LTFU).

**Methods:**

We conducted a retrospective cohort review of previously collected data in Fujian, Jiangxi, Zhejiang and Chongqing provinces in China from 1 June 2016–31 December 2017. The study population included all HBV-exposed infants and their mothers. SAS software was used for statistical analyses. Bivariate and multivariate regression analyses (presented in odds ratio [*OR*] with 95% confidence intervals [*CI*]) were used to compare the proportional differences of factors associated with PVST not being completed.

**Results:**

Among enrolled 8474 target infants, 40% of them transferred out of the study provinces without further information and 4988 were eligible for PVST. We found 20% (994) of infants were not compliant with the testing cascade: 55% of LTFU occurred because parents refused venous blood sample collection or failure of sample collection in the field, 16% transferred out after 6 months of age, and 10% of families chose to have independent, confidential PVST completed without reporting results. High PVST noncompliance rates were more likely to be from Fujian (a*OR* = 17.0, 95% *CI*: 9.7–29.9), Zhejiang (a*OR* = 5.7, 95% *CI*: 3.2–10.1) and Jiangxi (a*OR* = 1.9, 95% *CI*: 1.0–3.4), and from HBV e antigen positive mother (a*OR* = 1.2, 95% *CI*: 1.1–1.4).

**Conclusions:**

This study found that the LTFU rate reached 20% in PVST program, which was a significant problem. We recommend implementing a national electronic information system for tracking HBV at risk mother-infant pairs; encourage further research in developing a less invasive means of completing PVST, and take effective measures nationally to reduce HBV stigma. Without reducing the loss to follow up rate among infants eligible for PVST, elimination of vertical HBV transmission will be impossible.

**Electronic supplementary material:**

The online version of this article (10.1186/s40249-019-0568-y) contains supplementary material, which is available to authorized users.

## Multilingual abstracts

Please see Additional file 1 for translations of the abstract into the five official working languages of the United Nations.

## Background

Hepatitis B virus (HBV) is globally endemic, with approximately 257 million chronically infected and nearly 900 000 deaths per year [1, 2]. In 2016, the World Health Organization (WHO) issued the Global Health Sector Strategy on Viral Hepatitis 2016–2021, focused on eliminating viral hepatitis as a major public health threat by 2030 [3]. The prevention of mother-to-child transmission (PMTCT) is one of the five core areas of this strategic plan, aiming to achieve an HBV prevalence among children under five years to < 0.1% [3].

China is a highly-endemic country for HBV, with pre-vaccine era chronic HBV rates of approximately 10%, with the majority of infections secondary to vertical transmission during childbirth [4, 5]. In response, the Chinese government began providing the hepatitis B vaccine (HepB) for newborns free-of-charge. The government also mandated close collaboration between maternal and child healthcare (MCH) institutions and immunization departments, to promote timely birth dosing (TBD) of HepB within 24 h of delivery and improve completion rates of the three dose vaccination series [6].

In 2010, an integrated PMTCT (iPMTCT) programme for HIV, syphilis and hepatitis B was established with subsequent expansion throughout China by 2015 [7]. This programme provided free maternal HBV screening (HBsAg screening) and administration of the TBD and hepatitis B immunoglobulin (HBIG) for infants.

With those efforts, China has demonstrated significant achievements in HBV vertical transmission prevention, with the HBsAg prevalence in children < five years of age at 0.32% in 2014, a 97% reduction from the pre-vaccination era [8].

Despite remarkable success, it is estimated that there remains 16–18 million mothers who could give birth annually in China, nearly 6% of them with chronic HBV infection, and one-third with high HBV viral loads [9]. Consequently, more than 50 000 Chinese infants would likely acquire chronic HBV infection during birth yearly [10].

The post-vaccination serological test (PVST) for children born from HBsAg positive mothers is utilized to monitor the success or failure of the HepB vaccination series in at risk infants. The PVST is measured by venous sampling at 1–2 months after the third dose of HepB to determine whether HBV exposure is effectively prevented [2, 11, 12]. The WHO and Western Pacific Region emphasize this strategy because from a public health program perspective, PVST identifies program and strategy failures, allowing for improvement of both. Additionally, from individual perspective, it can help to confirm whether the infant is protected, remains susceptible and needs to be revaccinated, or is infected and needs referral to an appropriate healthcare provider.

Thus far, only a few developed countries have successfully implemented routine PVST for infants born at risk for HBV [11–14]. In the Western Pacific Region, where HBV is highly endemic, no country has demonstrated the ability to scale-up a PVST-based strategy for HBV prevention. In order to evaluate the feasibility, we coordinated with the WHO to take a first look at the implementation of a PVST pilot program in four provinces within China.

At risk infants who do not complete the PVST cascade have an increased danger of developing chronic HBV and associated sequela. In this study, our objectives are to 1) determine the proportion of infants who were lost to follow up (LTFU) within the PVST cascade and 2) potential reasons for LTFU, which could provide insight and guidance for future programming strategies and have significant healthcare policy implications.

## Methods

### Study design and setting

This was a retrospective cohort study utilizing previously collected programmatic data, following the Strengthening the Reporting of Observational Studies in Epidemiology (STROBE) guidelines for observational studies [15].

The PVST program had been implemented in Chongqing Municipality, Zhejiang, Jiangxi and Fujian provinces. In each province, among the urban districts and rural counties whose number of chronic HBV infected pregnancies screened in 2015 were higher than the provincial average level, we randomly selected one urban district and one rural county as research sites. Both of the district and county in Jiangxi Province carried out the PVST cascade through the local immunization system (IS), while in Zhejiang Province they carried out the PVST cascade through local MCH hospitals. In Chongqing and Fujian, the county chose to make the PVST cascade through IS and the district through MCH hospitals in each province.

In each county, the PVST program was organized by county CDC, the difference for IS and MCH was reflected in the details of blood collection. For IS supported counties, all infants get blood sampling and testing completed by the county-level CDC lab. For MCH supported counties, the county CDC cooperates with MCH hospitals, all the infants receive blood sampling and testing within MCH hospitals and the results are provided to county CDC. An enzyme-linked immunosorbent assay (ELISA) method was utilized for PVST testing in both of the IS and MCH laboratories and the reagent for HBV sero-markers was from the same manufacturer (Beijing Wantai Biological Pharmacy Enterprise Co. Ltd., Beijing, China).

### Study population

The target population in PVST grogram were HBV-exposed infants and their mothers, with the inclusion criteria requiring: 1) the infants were born during 1 Jun 2016–31 Dec 2017; 2) the mother was screened for HBsAg positive; 3) the guardian of infants consented to participate in the project.

### Data source and variables

Variables and data considered to be related to LTFU were extracted from the pilot PSVT program database utilizing a standardized data abstraction form. Data was double-entered into a dedicated database using EPI Data 3.1 (EPI Data Association, Odense, Denmark) and validated for consistency. The PVST database is SPSS-based (IBM Corp, Armonk, NY, USA) and kept by National Immunization program (NIP), Chinese Center for Disease Control and Prevention, the project SPSS database for 2016–2017 have been cleaned and validated by NIP data team.

Study variables included: mother’s age, parity, education, mothers’ HBV infected status, region, infant birthdate, infant hepatitis B vaccination information, date of PVST completion, reasons for not completing PVST. The definition for LTFU was:

1. refused or failure to obtain blood: the parents/guardians directly refused venous blood collection, or the nurses could not draw out blood sample;
2. transfer out: the child left the pilot county before a PVST could be completed;
3. request to delay blood testing; the parents/guardians wanted to delay PVST until after the child is one year old;
4. self-test for privacy: parents/guardians chose to do PVST testing in a different laboratory and the results would be kept by them without feedback to CDC;
5. wrong contact information: the cellphone number that the parents/guardians provided was incorrect and they could not be contacted;
6. others: included not reaching the PVST interval of at least one month after the third dose vaccination, or the parents had no time to take child to complete the PVST.

### Statistic analysis

SAS software (version9.4, SAS Institute, Inc., Cary, NC, USA) was used for statistical analyses. We utilized descriptive statistics for demographic data and the reasons for LTFU. Results were presented as frequency and proportions. Bivariate and multivariate regression analyses (presented in odds ratio [*OR*] with 95% confidence intervals [*CI*]) were used to compare the proportional differences of factors associated with PVST not been completed. The statistical significance was considered for those results with a two-tailed *P* <  0.05.

## Results

### Base information

**Fig. 1:**
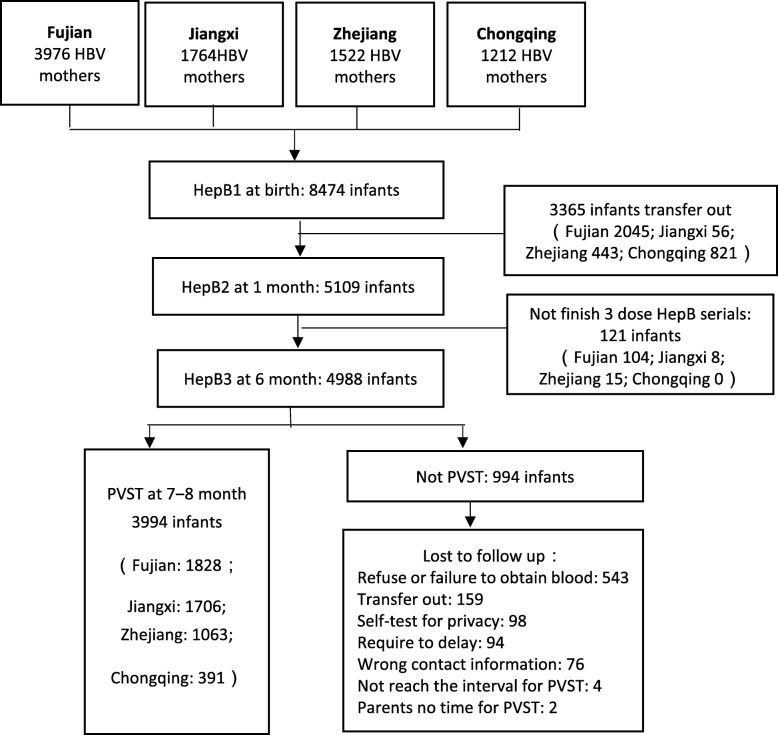
Four provinces with HBV infected mothers and their infants enrolled in PVST cascade, China, 2016–2017

There were 8474 infants and their mothers enrolled in the study; 3365 (40%) of the infants transferred out of their birth county after discharge from the hospital and no further data was available on them (see Fig. 1). Another 121 infants were excluded secondary to not completing the third hepatitis B vaccination dose or not meeting the minimum time interval following the third dose. Among the 4988 infants who met PVST criteria, 20% (994) did not complete the testing cascade (see Fig. 1).

### Demographic characteristics of LTFU infants

Of the 4988 eligible PVST infants’ mothers, 1828 (37%) were from Fujian, 1706 (34%) were from Jiangxi, and 1063 (21%) and 391 (8%) were from Zhejiang and Chongqing respectively (see Table 1). Among those HBsAg positive mothers, 1645 (33%) were HBV e antigen positive. There were 994 infants (20%) who did not receive serologic testing after the third dose of HepB vaccine. The noncompliance rates in Fujian, Jiangxi, Zhejiang and Chongqing were 38, 6, 17 and 4% respectively.

**Table 1:** The characteristic of mothers with HBV in four provinces, China, 2016–2017

	Fujian		Jiangxi		Zhejiang		Chongqing		Total	
	n	(%)	n	(%)	n	(%)	n	(%)	n	(%)
Age group (years old)										
15–24	174	(10)	576	(34)	112	(11)	100	(26)	962	(19)
25–29	787	(43)	748	(44)	411	(39)	163	(42)	2109	(42)
30–34	608	(33)	274	(16)	326	(31)	83	(21)	1291	(26)
35–49	259	(14)	108	(6)	214	(20)	45	(12)	626	(13)
Education										
Primary school and lower	451	(25)	186	(11)	79	(7)	15	(4)	731	(15)
Middle hool	1224	(67)	1285	(75)	565	(53)	259	(66)	3333	(67)
Bachelor degree and above	153	(8)	235	(14)	419	(39)	117	(30)	924	(19)
Parity										
1	490	(25)	593	(11)	305	(7)	166	(4)	1554	(15)
≥ 2	1338	(67)	1113	(75)	758	(53)	225	(66)	3434	(67)
HBV e antigen										
Negative	1131	(62)	1225	(72)	515	(49)	239	(61)	3110	(63)
Positive	664	(36)	337	(20)	498	(47)	146	(37)	1645	(33)
Not test	33	(2)	144	(8)	50	(5)	6	(2)	233	(5)
Total	1828	(100)	1706	(100)	1063	(100)	391	(100)	4988	(100)

### Potential reasons for LTFU

**Fig. 2:**
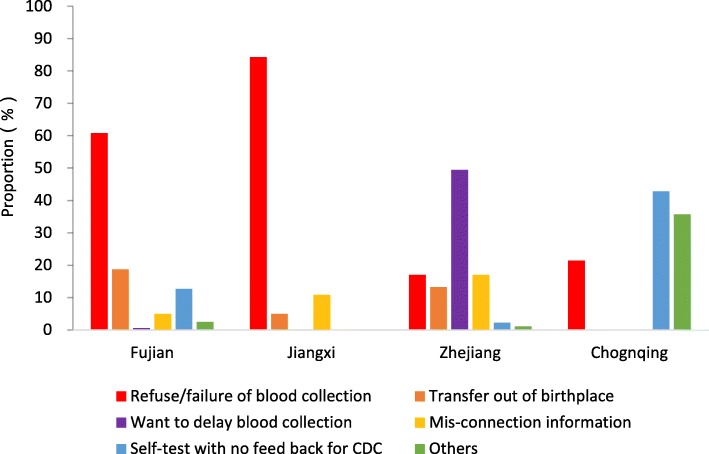
Proportion of non-compliance reasons in four provinces, China, 2016–2017

Among children who did not complete PVST, 55% were because parents refused venous blood sample collection or failure of sample collection in the field, 16% were due to transferring out after 6 months old, and 10% were due to their parents wanting to delay serological testing to 12 months or later (see Fig. 2). There were 98 (10%) infants who received PVST without reporting results through families seeking independent, confidential testing. Lastly, 76 infants (8%) were LTFU for providing wrong contact information.

Infant venous blood sample collecting difficulties accounted for 61% in Fujian and 84% in Jiangxi of PVST noncompliance. In Zhejiang, nearly 50% of PVST noncompliance occurred to parents wanting to delay the test. Finally, for PVST noncompliant in Fujian, infant immigration from birthplace with their parents was 19%.

In the multivariable analysis, high PVST noncompliance rates were more likely to be from Fujian (a*OR* = 17.0, 95% *CI*: 9.7–29.9), Zhejiang (a*OR* = 5.7, 95% *CI*: 3.2–10.1) and Jiangxi (a*OR* = 1.9, 95% *CI*: 1.0–3.4), and from HBV e antigen positive mother (a*OR* = 1.2, 95% *CI*: 1.1–1.4), see Table 2. No statistically significant differences were seen among mothers’age, education or parity.

**Table 2:** Risk factors associated with PVST not being completed in four provinces, China, 2016–2017

	No. of observations	Non-compliance		Crude analysis		Multivariable analysis	
		n	%	OR (95% CI)	*P*-value	aOR (95% CI)	*P*-value
Province							
Fujian	1828	696	38.1	0.1 (0.0–0.1)	< 0.001	17.0 (9.7–29.9)	< 0.001
Jiangxi	1706	102	6.0	0.6 (0.3–1.0)		1.9 (1.0–3.4)	
Zhejiang	1063	182	17.1	0.2 (0.10–0.3)		5.7 (3.2–10.1)	
Chongqing	391	14	3.6	Reference		Reference	
Age group of mothers (years old)							
15–24	962	124	12.9	2.0 (1.5–2.6)	< 0.001	1.1 (0.8–1.6)	0.90
25–29	2109	409	19.4	1.2 (1.0–1.5)		1.0 (0.8–1.3)	
30–34	1291	319	24.7	0.9 (0.7–1.1)		1.1 (0.8–1.4)	
35–49	626	142	22.7	Reference		Reference	
Education of mother							
Primary school and lower	731	208	28.5	2.3 (1.8–2.9)	< 0.001	1.2 (0.9–1.6)	0.54
Middle school	3333	649	19.5	1.4 (1.1–1.7)		1.1 (0.9–1.4)	
Bachelor degree and above	924	137	14.8	Reference		Reference	
Parity of mother							
1	1554	254	16.3	0.7 (0.6–0.8)	< 0.001	0.8 (0.7–1.0)	0.21
≥ 2	3434	740	21.5	Reference		Reference	
HBV e antigen of mother^a^							
Positive	1645	397	24.1	1.5 (1.2–1.6)	< 0.001	1.2 (1.1–1.4)	< 0.001
Negative	3110	573	18.4	Reference		Reference	
Gender of children							
Male	2657	535	20.1	1.0 (0.9–1.2)	0.72	–	–
Female	2331	459	19.7	Reference		–	
Total	4988	994	19.9	–	–	–	–

^a^ missing 233 for HBV e antigen un-testing; − Not applicable

## Discussion

Our study found that LTFU within the PVST cascade was 20% in the four study provinces and varied from 4% in Chongqing to 38% in Fujian. The main reasons for LTFU among infants completing all three HepB doses were because parents either refused venous blood sample collection or there was a failure of sample collection in the field, which accounted for more than 50% of LTFU. We also found there was a higher risk for LTFU among those mothers who were HBV e antigen positive. Lastly, transferring out of the study provinces accounted for losing follow up data on a large proportion (40%) of the total infant cohort.

This study is the first look within China focusing on the feasibility of a PVST cascade, the frequency of LTFU and factors associated with LTFU. Of the 20% of families not compliant with the PVST cascade, the highest proportion were found to be in Fujian Province (38%). Among previous studies, LTFU rates for PVST were greater than 20% in United States, while in United Kingdom the LTFU rate reported was approximately 10% [11, 13]. Thus, even in more developed countries, LTFU for PVST among infants at high risk for developing chronic HBV infected remains significant.

After Fujian Province, we found the LTFU rate was highest in Zhejiang (17%), and Jiangxi (6%) while Chongqing had the lowest rate (4%). In addition, besides those infants with clear immunization information but being LTFU, there were also 68% in Chongqing, 51% in Fujian, 32% in Jiangsu and 29% in Zhejiang of initially enrolled infants in hospitals who could not be followed up due to changing their living areas. This widely varied LTFU rate might also be related to the migration of families within these particular provinces. Based upon the sixth national census, population migration was reported at 17% within China, with the primary flow moving from the Central and Western provinces and an increase in migration towards the East provinces and other more economically developed areas [16]. Chongqing, located in Southwest of China, had the higher proportion of movement of families to other regions while Fujian and Zhejiang provinces, located in East area, reported the higher newly registered inhabitants. This is consistent with our observation that there was a higher infant transferred out rate in Chongqing than the other three provinces.

The second reason for Fujian and Jiangxi having higher LTFU rates was likely because of the highly HBV endemic context within these provinces. The HBsAg prevalence was more than 15% in Fujian and 13% in Jiangxi [5]. The large number of PVST target infants possibly made the implementation strategy logistically more complex.

In China, maternal HBV screening and infant vaccination results are registered in separate electronic data systems, which belong to the MCH and NIP. There is currently no cross-platform information sharing capabilities between these two systems. Additionally, there were limitations in the ability to share electronic data regarding infant vaccinations between provinces. Therefore, movement of families within different provinces makes PVST care continuity challenging. Establishment of a national information system with integrated children vaccination information and maternal screening at the national level, would likely significantly improve PVST implementation, improve follow up and ultimately lead to a reduction in the frequency of HBV vertical transmission.

We found that a key factor for LTFU was the difficulty of venous blood sample collection from infants, which accounted for more than 50% of PVST noncompliance. Another recent study found that only two thirds of infants eligible for PVST were successful with completing the necessary venous blood testing [17]. Although using venous blood to test HBV sero-markers by ELISA would get accurate results [18], it is currently unsuitable for scaled-up monitoring for routine work out of hospital.

Venous sampling of infants can be challenging and requires a skill set that is not easily achieved. Consequently, parental emotions around infant blood draws, especially difficult ones, can impede success. Alternative methods of blood sample collection and detection, such as using a finger stick source for an HBV rapid test [19], are crucial to improving PVST compliance going forward.

We also found those infants from HBV e antigen positive mothers had a higher risk of LTFU than those from HBV e antigen negative mothers. This might be due to the HBV stigma existing within China [20–23]. In 2010, the Ministry of Health together with Ministry of Education mandated that children cannot be limited to school or nursery for being HBsAg positive [24]. Regardless, some parents remain concerned about testing results being known by others, which could affect children’s future study and life. This stigma is especially challenging for HEAP mothers because they have a higher risk of HBV vertical transmission [2]. Additionally, 10% of the noncompliant infants in our study completed PVST independently, without providing feedback about the testing results, which supports the theory of stigma impact of parental decision making.

## Strengths & limitations

The strength of our study was the accuracy of data, which was based on a high-quality database obtained from a prospectively designed study on PVST. Information on each mother-infant pair was recorded and validated, which reduces the risk of error and bias. This study had two primary limitations. First, 40% of the PVST mother-infant pairs transferred out of the study areas and there was no further information available on them. Secondly, our study data was from only four of 34 provinces within China, which may not be representative for whole country.

## Conclusions

This study found that LTFU is a significant problem with implementation of a PVST program. Until the LTFU rate can be reduced, achieving further meaningful reductions in the vertical transmission of HBV will remain challenging. We recommend implementing a national electronic information system for tracking HBV at risk mother-infant pairs. We also strongly encourage further research in developing a less invasive means of completing post-vaccination serological testing for HBV. Lastly, stigma surrounding HBV infection remains and needs to be addressed collaboratively to reduce this unnecessary burden by the Nation Health Commission and others.

## Additional file

Additional file 1: Multilingual abstracts in the five official working languages of the United Nations. (PDF 616 kb)

## Data Availability

The dataset used and analyzed during the current study is available from the corresponding author on reasonable request.

## Abbreviations

CI: Confidence intervals
ELISA: Enzyme-linked immunosorbent assay
HBIG: Hepatitis B immunoglobulin
HBV: Hepatitis B virus
HepB: Hepatitis B vaccine
iPMTCT: Integrated PMTCT
IS: Immunization system
LTFU: Lost to follow up
MCH: Maternal and child healthcare
*OR*: Odds ratio
PMTCT: Prevention of mother-to-child transmission
PVST: Post-vaccination serological testing
TBD: Timely birth dosing
WHO: World Health Organization
