# Supplementary material for: Compliance among infants exposed to hepatitis B virus in a post-vaccination serological testing program in four provinces in China
Source: Infect Dis Poverty. 2019 Jul 4;8:57. doi: 10.1186/s40249-019-0568-y (PMC6610824; doi:10.1186/s40249-019-0568-y)
Supplement: Supplementary file 1 — Multilingual abstracts in the five official working languages of the United Nations. (PDF 616 kb) [file 40249_2019_568_MOESM1_ESM.pdf]

Translation of the abstract into the five official working languages of the United Nations

الامتثال بين الرُضع المعرضين لخطر الإصابة بالتهاب الكبد الوبائي ب في برنامج الاختبارات المصلية بعد التطعيم بأربعة مقاطعات في الصين

هوى تشنغ ، قوه مين تشانغ ، بو لين تشان ، فو تشن وانغ ، لانس إيفريت رودوالد ، نينغ مياو ، شياو جين صن ، زون دونغ يين ، جيفري إدواردز ، هوا تشينغ وانغ

ملخص

المعلومات الأساسية: ما يزال انتقال فيروس التهاب الكبد ب (HBV) من الأم إلى الطفل أكثر صور انتقال عدوى HBV شيوعًا في الصين. وتتضمن إجراءات منع الانتقال العمودي للعدوى إعطاء سلسلة لقاح التهاب الكبد ب (HepB) الكاملة والجلوبولين المناعي لالتهاب الكبد ب في الوقت المناسب. تُستخدم الاختبارات المصلية بعد التطعيم (PVST) لتحديد حالة الرضيع بعد التعرض لفيروس HBV وإكمال سلسلة series. ونحن نهدف إلى تحديد معدل الامتثال إلى سلسلة اختبارات PVST الخاصة بالأمهات المصابات بفيروس HBV وإلى تحليل العوامل المرتبطة بالطفل الذي تخلف عن المتابعة (LTFU).

الأساليب: لقد قمنا بمراجعة حشدية رجعية الأثر لبيانات تم جمعها سابقًا في مقاطعات فوجيان وجيانغسو وتشجيانغ وتشونغتشينغ في الصين من يونيو 2016 وحتى ديسمبر 2017. تضمنت الفئة الخاضعة للدراسة جميع الرضع المعرضين لفيروس HBV وأمهاتهم. وتم استخدام برنامج SAS لإجراء التحليلات الإحصائية. وتم استخدام تحليل انحدار ثنائي المتغير ومتعدد المتغيرات (في صورة نسبة للاحتمالات [OR] مع فواصل ثقة 95% [CI]) للمقارنة بين الاختلافات التناسبية للعوامل المرتبطة بعدم إكمال اختبارات PVST.

النتائج: بين 8474 رضيعًا مستهدفًا مسجلًا، 40% منهم انتقلوا إلى خارج المقاطعات التي أجريت فيها الدراسة دون توفر معلومات إضافية و4988 كانوا مؤهلين لاختبارات PVST. ووجدنا أن 20% (994) كانوا غير ممتثلين لسلسلة الاختبارات: 55% تخلفوا عن المتابعة (LTFU) لأن أولياء أمرهم رفضوا جمع عينة دم وريدية أو بسبب فشل إجراء سحب العينة في المعمل، و6% انتقلوا خارج المقاطعات بعد أن بلغوا من العمر 6 أشهر، و0% من الأسر اختاروا إجراء اختبارات LTFU بشكل سري ومستقل دون الإبلاغ عن النتائج. ومن المرجح أن تكون أعلى نسب عدم الامتثال لاختبارات PVST من فوجيان (OR =7.0، 95% CI: 9.7–29.9)، وتشجيانغ (OR = 5.7، 95% CI: 3.2–10.1) وجيانغسو (OR =.9، 95% CI:.0–3.4)، ومن الأم إيجابية مستضد غلاف فيروس e HBV (OR =.2، 95% CI:.1–1.4).

الاستنتاجات: توصلت هذه الدراسة إلى أن نسبة التخلف عن المتابعة (LFTU) قد بلغت 20% في برنامج PVST، الأمر الذي مثّل مشكلة كبيرة. ونوصي بتنفيذ نظام معلومات إلكتروني وطني خاص بتتبع ثنائيات الأم-الرضيع المعرضة لخطر فيروس HBV؛ وبتشجيع إجراء أبحاث إضافية لتطوير وسائل أقل توغلًا لإكمال برنامج PVST وباتخاذ إجراءات فعالة على مستوى الدولة للحد من وصمة فيروس HBV. دون تخفيض نسبة التخلف عن المتابعة بين الرضع المؤهلين لبرنامج PVST، سيكون القضاء على الانتقال العمودي لعدوى فيروس HBV مستحيلًا.

Translated from English version into Arabic by Israa Abdelkarim, proofread by Ahmed Ibrahim, through

TRANSLATORS WITHOUT BORDERS

中国四省乙肝病毒感染高风险儿童疫苗免疫后血清学监测的依从性研究

郑徽，张国民, Po-Lin Chan，王富珍, Lance Everett Rodewald，缪宁，孙校金，尹遵栋, Jeffrey Edwards，王华庆

摘要

引言：乙肝病毒（HBV）的母婴传播仍是中国 HBV 感染的主要方式。HBV 感染母亲所生儿童及时接种乙肝疫苗和乙肝免疫球蛋白可以有效预防 HBV 母婴垂直传播。对 HBV 高暴露风险儿童开展疫苗免疫后的血清学监测（PVST）是评价疫苗免疫效果的重要途径。本研究旨在了解 HBV 感染母亲所生新生儿对 PVST 的依从性，并分析造成 PVST 失访的影响因素。

方法：我们对 2016 年 6 月 1 日-2017 年 12 月 31 日在福建、江西、浙江和重庆四省（直辖市）开展的 PVST 监测项目数据库进行了回顾性分析。研究对象包括项目地区内研究期间发现的所有 HBV 感染母亲及所生儿童。使用 SAS 软件对造成儿童 PVST 不依从的因素开展双变量和多变量回归分析，结果以优势比[OR]和 95%置信区间[CI])表示。
结果：本研究共入组 8474 名目标儿童，其中 40%的儿童因出生后离开项目地区造成信息不全剔除。在 4988 名合格的 PVST 儿童中，我们发现 20%（994 人）对 PVST 监测不依从，其中 55%儿童是因为静脉血采集困难或现场采血失败、16%儿童在 6 月龄后随父母迁出项目地区、10%儿童家长选择自行去医疗机构检测且结果保密。多因素分析显示，福建(a*OR* = 17.0, 95% *CI*: 9.7–29.9)、浙江(a*OR* = 5.7, 95% *CI*: 3.2–10.1)、江西(a*OR* = 1.9, 95% *CI*: 1.0–3.4)和母亲 HBV e 抗原阳性是影响儿童 PVST 不依从性升高的因素。
结论：本研究发现在 PVST 监测项目中的儿童失访（20%）是一个值得关注的问题。建议全国建立覆盖妇幼和免疫规划的统一电子信息系统，以识别并跟踪 HBV 感染母亲及所生儿童；进一步研究低侵入性的 PVST 标本采集及检测的方法；在全国范围内采取有效措施减少社会对 HBV 感染者的歧视。如果不降低 PVST 目标儿童的失访率，将很难实现 HBV 的垂直传播消除。

Translated from English version into Chinese by Hui Zheng

Observance parmi des nourrissons exposés au virus de l'hépatite B dans le cadre d'un programme de tests sérologiques post-vaccination dans quatre provinces de Chine

Hui Zheng, Guo-min Zhang, Po-lin Chan, Fu-zhen Wang, Lance Everett Rodewald, Ning Miao, Xiao-jin Sun, Zun-dong, Yin, Jeffrey Edwards, Hua-qing Wang

RÉSUMÉ

Contexte: La transmission mère-enfant du virus de l'hépatite B (VHB) reste le mode d'infection le plus fréquent en Chine. La prévention de la transmission directe du VHB nécessite l'administration en temps utile de la série complète d'injections du vaccin contre l'hépatite B et d'immunoglobulines anti-hépatite B Un test sérologique post-vaccination (TSPV) est utilisé pour déterminer le statut d'un nourrisson exposé au VHB après sa vaccination complète contre l'hépatite B. Notre but était de déterminer la fréquence de l'observance par les mères infectées par le VHB d'une cascade de tests TSPV et d'analyser les facteurs associés aux nourrissons perdus de vue.
Méthodes: Nous avons mené une étude de cohorte rétrospective sur des données relevées antérieurement dans les provinces chinoises du Fujian, du Jiangxi, du Zhejiang et du Chongqing, entre juin 2016 et décembre 2017. La population à l'étude était constituée des nourrissons exposés au VHB ainsi que de leurs mères. Le logiciel SAS a été utilisé pour les analyses statistiques. Des analyses de régression bivariées et multivariées (présentées sous forme de rapport de cotes [*OR*] avec des intervalles de confiance [*IC*] à 95 %) ont été utilisées pour comparer les différences proportionnelles des facteurs associés aux TSPV qui n'ont pas été effectués.
Résultats: Sur les 8474 nourrissons ciblés inclus dans l'étude, 40 % ont quitté les provinces de l'étude et aucune autre information n'était disponible à leur sujet et 4988 étaient éligibles pour le TSPV. La cascade de tests spécifiée n'a pas été respectée pour 20 % (994) des nourrissons :55 % des enfants perdus de vue l'ont été parce que les parents ont refusé les prélèvements d'échantillons de sang veineux ou parce que la collecte des échantillons sur le terrain n'a pas pu être menée à bien ; 6 % des nourrissons ont quitté la province après l'âge de 6 mois et 0 % des familles ont choisi de faire réaliser un TSPV indépendant et confidentiel sans rendre compte des résultats. Les taux de non-observance du TSPV tendaient à être plus élevés dans le Fujian (a*OR* = 7,0, *IC* à 95 % de 9,7 à 29,9), le Zhejiang (a*OR* = 5,7, *IC* à 95 % de 3,2 à 10,1) et le Jiangxi (a*OR* = 9, *IC* à 95 % de 0 à 3,4), et également lorsque la mère était positive pour l'antigène HBE (a*OR* = 2, *IC* à 95 % de 1 à 1,4).

Conclusions: Cette étude a révélé un taux de nourrissons perdus de vue dans le programme de TSPV qui atteignait 20 %. Le problème est grave et nous recommandons de mettre en place un système national d'information électronique pour suivre les dyades mère-enfant à risque d'infection par le VHB, d'encourager la poursuite des recherches pour mettre au point d'un moyen moins invasif de tester la sérologie post-vaccination et de prendre des mesures efficaces à l'échelle nationale pour réduire la stigmatisation liée à l'hépatite B. Il ne sera pas possible d'éliminer la transmission verticale du VHB si le taux de nourrissons perdus de vue au contrôle de leur sérologie post-vaccination n'est pas réduit.

Translated from English version into French by Marina Della Torre, proofread by Suzanne Assenat, through

TRANSLATORS
WITHOUT BORDERS

Приверженность детей, подвергшихся воздействию вируса гепатита В, программе серологического тестирования после вакцинации в четырех провинциях Китая

Хуэй Чжэн, Го-мин Чжан, По-лин Чан, Фу-чжэнь Ван, Ланс Эверетт Родевальд, Нин Мяо, Сяо-Цзинь Сунь, Цзун-дон Инь, Джеффри Эдвардс, Хуа-цин Ван

АННОТАЦИЯ

Справочная информация: передача вируса гепатита В (ВГВ) от матери к ребенку остается наиболее распространенным путем передачи инфекции ВГВ в Китае. Профилактика вертикальной передачи ВГВ включает своевременное введение полной серии вакцин и иммуноглобулина против гепатита В. Серологическое тестирование после вакцинации (СТПВ) используется для определения статуса младенца после воздействия ВГВ и завершения серии вакцин. Наша цель - определить частоту приверженности каскада СТПВ среди инфицированных вирусом гепатита В матерей и проанализировать факторы, связанные с потерей ребенка для последующего наблюдения (ПРПН).

Методы: Мы провели ретроспективное когортное исследование ранее собранных данных в провинциях Фуцзянь, Цзянси, Чжэцзян и Чунцин в Китае с июня 2016 г. по 31 декабря 2017 г. Исследуемая группа включала всех младенцев, подвергшихся воздействию ВГВ, и их матерей. Программное обеспечение SAS использовалось для статистического анализа. Двумерный и многомерный регрессионный анализ (представленный в отношении вероятностей [*ОВ*] с 95% доверительным интервалом [*ДИ*]) использовался для сравнения пропорциональных различий в факторах, связанных с незавершенными СТПВ.

Результаты: Среди зарегистрированных 8474 целевых детей, 40 % были исключены из исследования провинций без дополнительной информации, а 4988 соответствовали критериям для СТПВ. Мы обнаружили, что 20 % (994) детей не соблюдали каскад тестирования: 55 % ПРПН произошли из-за того, что родители отказались от взятия пробы венозной крови или от взятия пробы в полевых условиях, 6 % были исключены после 6-месячного возраста и 0 % семей решили пройти независимое, конфиденциальное СТПВ без сообщения результатов. Высокие показатели несоблюдения СТПВ чаще встречались в Фуцзяни (а*ОВ* = 7,0, 95 % *ДИ*: 9,7–29,9), Чжэцзяне (а*ОВ* = 5,7, 95 % *ДИ:* 3,2–10,1) и Цзянси (а*ОВ* =. 9, 95 % *ДИ*: 0,0–3,4), а также от ВГВ е-антиген-положительной матери (а*ОВ* = 0, 2, 95 % *ДИ*: 0,1–1,4).

Выводы: Это исследование показало, что показатель ПРПН достиг 20 % в программе СТПВ, что оказалось значительной проблемой. Мы рекомендуем внедрить национальную электронную информационную систему для отслеживания ВГВ в парах «мать-младенец»; поощрять дальнейшие исследования в разработке менее инвазивных средств для завершения СТПВ и принимать эффективные меры на национальном уровне для снижения стигмы ВГВ. Без снижения количества случаев потери наблюдения детей, соответствующих критериям СТПВ, устранение вертикальной передачи ВГВ будет невозможным.

Translated from English version into Russian by Anna Kukharchuk, proofread by Michael Orlov, through

TRANSLATORS WITHOUT BORDERS

El cumplimiento entre bebés expuestos al virus de la hepatitis B en un programa de pruebas serológicas posteriores a la vacunación en cuatro provincias de China

Hui Zheng, Guo-min Zhang, Po-lin Chan, Fu-zhen Wang, Lance Everett Rodewald, Ning Miao, Xiao-jin Sun, Zun-dong Yin, Jeffrey Edwards, Hua-qing Wang

RESUMEN

Antecedentes: la transmisión del virus de la hepatitis B (VHB) de madre a hijo sigue siendo la forma más común de infección por el VHB en China. La prevención de la transmisión vertical del VHB implica la administración oportuna de la serie completa de vacunas contra la hepatitis B (HepB) y la inmunoglobulina contra la hepatitis B. Las pruebas serológicas posteriores a la vacunación (PVST, por sus siglas en inglés) son utilizadas para determinar el resultado de un bebé después de la exposición al VHB y la finalización de la serie HepB. Nuestro objetivo es determinar la frecuencia de cumplimiento con PVST en cascada para las madres infectadas por el VHB y analizar los factores asociados con los bebés perdidos durante el seguimiento (LTFU).

Métodos: llevamos a cabo una revisión de cohortes retrospectiva de los datos recopilados anteriormente en las provincias de Fujian, Jiangxi, Zhejiang y Chongqing en China de junio de 2016 al 31 de diciembre de 2017. La población del estudio incluyó a todos los bebés expuestos al VHB y sus madres. El software SAS se utilizó para los análisis estadísticos. Se usaron análisis de regresión bivariables y multivariables (presentados en razón de probabilidades [*OR*] con intervalos de confianza del 95 % [*IC*]) para comparar las diferencias proporcionales de los factores asociados con que no se completaran las PVST.

Resultados: de los 8474 bebés objetivo inscritos, el 40 % de ellos fueron transferidos fuera de las provincias del estudio sin más información y 4988 fueron elegibles para las PVST. Encontramos que el 20 % (994) de los bebés no cumplía con la prueba en cascada: el 55 % de LTFU ocurrió porque los padres rechazaron la toma de muestras de sangre venosa o debido al fracaso de la recolección de muestras en el campo, el 6 % se transfirió después de los 6 meses de edad y el 0 % de las familias eligió completar las PVST de manera independiente y confidencial sin informar los resultados. Las altas tasas de incumplimiento de las PVST eran más propensas a ser de Fujian (a*OR* = 7,0, *CI* del 95 %: 9,7–29,9), Zhejiang (a*OR* = 5,7, *CI* del 95 %: 3,2–10,1) y Jiangxi (a*OR* = 0,9, *CI* del 95 %: 0,0–3,4), y de la madre positiva para el antígeno e del VHB (a*OR* = 0,2, *CI* del 95 %: 0,1–1,4).

Conclusiones: este estudio encontró que la tasa de LTFU alcanzó el 20 % en el programa de PVST, lo cual fue un problema significativo. Recomendamos implementar un sistema nacional de información electrónica para el seguimiento de las parejas de madres y bebés con VHB en riesgo; fomentar nuevas investigaciones para desarrollar un medio menos invasivo para completar las PVST y tomar medidas efectivas a nivel nacional para reducir el estigma del VHB. Sin reducir la tasa de pérdidas de seguimiento entre los bebés elegibles para las PVST, la eliminación de la transmisión vertical del VHB será imposible.

Translated from English version into Spanish by Ignacio Montalvo, proofread by Mayra León, through

TRANSLATORS
WITHOUT BORDERS
